# Layer-Specific Strain Analysis in Patients with Dilated Cardiomyopathy

**DOI:** 10.3390/biomedicines13010011

**Published:** 2024-12-25

**Authors:** Despina-Manuela Toader, Alina Paraschiv, Georgică Târtea, Gabriela Tiucu, Mihai Chițu, Raluca Stănișor, Oana Mirea

**Affiliations:** 1 EuroEchoLab, Craiova Cardiology Center, Emergency Hospital Craiova, 200642 Craiova, Romania; mihai_chitu98@yahoo.com (M.C.); stanisor_1992@yahoo.com (R.S.); oana.mirea83@gmail.com (O.M.); 2 Filantropy Hospital Craiova, 200143 Craiova, Romania; paraschivalina30@yahoo.com; 3 Interventional Cardiology, Cardiology Department, Emergency Hospital Craiova, 200642 Craiova, Romania; georgica.tartea@umfcv.ro; 4 Centre Hospitalier Sud Francilien, 91100 Corbeil Essonnes, France; gtiucu@yahoo.com; 5 Faculty of Medicine, University of Medicine and Pharmacy Craiova, 200349 Craiova, Romania

**Keywords:** dilated cardiomyopathy, layer-specific strain analysis, outcome

## Abstract

Background/Objectives: This study aimed to evaluate layer-specific strain according to etiology and assess whether subtle changes in longitudinal and circumferential layer strain are involved in predicting cardiac mortality during a two-year follow-up in patients with dilated cardiomyopathy admitted with heart failure decompensation. Methods: 97 patients with dilated cardiomyopathy and a left ventricle ejection fraction ≤ 40% were recruited, 51 with ischemic and 46 with nonischemic etiologies. Conventional and two-dimensional speckle-tracking echocardiography (2D-STE) were conducted in dilated cardiomyopathy patients with a compensated phase of heart failure before discharge. Layer-specific longitudinal and circumferential strain was assessed from the endocardium, mid-myocardium, and epicardium by two-dimensional (2D) speckle-tracking echocardiography. The gradient between the endocardium and epicardium was calculated. Results: Patients with nonischemic etiology of dilated cardiomyopathy presented smaller values of global and layer strain than patients in the ischemic group. GLS, GLSend, GLSend-GLSepi, CSPMend, CSPMend-CSPMepi, CSAP, CSAPend, and CSAPend-CSAPepi were the parameters with statistically significant decreased values in non-survivors compared with survivors. In multivariate analysis, only CSPMend showed an independent value in predicting mortality at two-year follow-up. Receiver operator curve analysis provided CSPMend of −10.8% as a cut-off value with a sensitivity of 80% and specificity of 61.05% in identifying the dilated cardiomyopathy and heart failure patients with a risk of death at two-year follow-up. Conclusions: GLS, GCS, and layer-specific strain analysis showed decreased values in nonischemic compared with ischemic dilated cardiomyopathy and also in non-survivors compared with survivors. CSPMend was the most sensitive strain parameter to identify patients with increased mortality risk at two-year follow-up.

## 1. Introduction

Dilated cardiomyopathy (DCM) is characterized by the left ventricular (LV) or biventricular dilation and systolic dysfunction without abnormal loading conditions such as valvular heart disease, arterial hypertension, or coronary artery disease severe enough to explain this dysfunction [1,2]. DCM is a cardiac disease with diffuse reactive interstitial fibrosis due to increased collagen proliferation of myofibroblasts and histopathological changes [3]. Despite progress in heart failure (HF) treatment, patients with DCM in advanced stages have an unfavorable prognosis, with mortality still being high. Until now, except for LV ejection fraction (LVEF), there are no echocardiographic parameters to identify the negative outcome precisely. Conventional echocardiography is recommended for evaluating DCM patients [4]. Because of the complex structure of the myocardial wall, EF is not a sufficient parameter to assess cardiac performance [5]. Therefore, assessing the cardiac function appropriately with more sensitive echocardiographic techniques may enhance the outcome prediction. Speckle-tracking echocardiography (STE) techniques provide incremental information in different clinical settings, including DCM. LV longitudinal strain (GLS) determined by two-dimensional speckle-tracking echocardiography (2D-STE) is a more accurate technique for assessing myocardial performance [6]. In addition to GLS, layer-specific strain (LSS) analysis assesses each myocardial layer separately [7] and allows differentiation between the active myocardial segmental deformation and the passive displacement of fibrotic segments [8]. The evaluation of DCM strain analysis is of particular interest because global strain parameters correlate with EF [9]. Circumferential strain (CS) and LSS analysis provided by STE might be as helpful as LS and could predict adverse outcomes in patients with DCM and advanced stages of HF. The study aims are as follows: (1) to characterize the features of LS, CS, and myocardial layer-specific strain in patients with DCM and advanced stages of HF according to etiology; (2) to find if some of these parameters could indicate adverse outcomes.

## 2. Materials and Methods

### 2.1. Study Population and Design

The present study was an observational prospective study conducted in a hospital setting on DCM patients admitted with signs and symptoms of HF. These patients were referred to our laboratory for clinically indicated 2D transthoracic echocardiography (2DTTE). Inclusion criteria consisted of confirmed DCM diagnosis, as per current guidelines, and giving consent to be part of the study. All the patients were evaluated in a compensated state before discharge to diminish the load-dependency effect on several echocardiographic parameters as much as possible. This case–control prospective research included 120 consecutive DCM patients admitted with decompensation of HF between January 2021 and December 2023 in the Department of Cardiology of the Emergency County Hospital of Craiova. DCM diagnosis was sustained by 2DTTE according to the last guidelines for chamber quantification [4]. The participants were aged 31 to 80 years (mean age 59.16 ± 20.14). Standard biochemical tests were performed at admission and throughout the hospital stay: hemoglobin, glycated hemoglobin, urea, creatinine, potassium, sodium, LDL, HDL cholesterol, and triglycerides. The Modification of Diet in Renal Disease (MDRD) formula was utilized to determine the predicted glomerular filtration rate [10]. Exclusion criteria were cardiogenic shock, hypervolemic status, severe malignant arrhythmias, advanced atrioventricular block, uncontrolled heart rate interfering with a proper echocardiographic acquisition, a suboptimal echocardiographic window, and patients or families not agreeing to participate. A total 23 patients presented exclusion criteria. The final cohort comprised 97 DCM patients and was divided into two groups: ischemic and nonischemic. The documented history of myocardial infarction, the presence of coronary artery disease confirmed by more than one vessel with luminal narrowing ≥ 70% at angiography, a history of percutaneous interventions (PCI), or coronary artery bypass grafting (CABG) sustained ischemic etiology. All patients underwent comprehensive transthoracic 2DTTE. Heart rate, blood pressure, height, and weight were measured before the echocardiographic image acquisition. Body surface area (BSA) was calculated using the DuBois formula [11]. Care was taken for the correct evaluation of the compensation state of HF to diminish as much as possible the load-dependency effect on echocardiographic parameters. Measurements were averaged from three cardiac cycles in sinus rhythm (SR) patients and five cardiac cycles in patients with atrial fibrillation (AF). Each patient signed the consent form for participation in the study. According to current guidelines, the patients were under medical treatment: beta blockers function of heart rate, sacubitril/valsartan (maximum tolerated dose), sodium–glucose cotransporter2 inhibitors, aspirin/anticoagulants, diuretics, and satins when appropriate. The study was compiled with the Declaration of Helsinki and approved by the Local Committees for Medical and Health Research Ethics.

### 2.2. Echocardiography

#### 2.2.1. Two-Dimensional Echocardiography

After compensation for hydric retention, 2DTTE images were acquired in all patients before discharge using commercial ultrasound systems (Vivid E95, GE, Vingmed, Horten, Norway). The analysis was performed offline using EchoPAC version 204 software (GE Vingmed Ultrasound, Horten, Norway). Standard Mmode, 2D, color, pulsed, and continuous wave Doppler images were acquired and stored digitally for offline analysis (EchoPac 204; GE Medical Systems, Horten, Norway). We recorded all echocardiographic measurements using standard views, avoiding foreshortening, and following the recommendations for chamber quantification [4]. Subjects with poor image quality (poor delimitation of >3 segments) were excluded from the study. We acquired LV apical 4-chamber, 3-chamber, 2-chamber views; long axis views; and short axis views at the mitral valve (MV), papillary muscles (PM), and apical level, with the patient lying in the left lateral decubitus position during a breath hold. Apical segments were the most apical region of the left ventricle that could be scanned, far behind the papillary muscles, from the parasternal short-axis view. Scanning the most apical segments for strain measurements was essential because this measurement might affect the results [5]. The gain and compression were adjusted to minimize the dropout of the LV endocardial and epicardial borders. The depth and sector angle were adjusted to include the entire LV, maintaining the frame rate between 40 and 60 frame/s. LV ejection fraction (LVEF) was calculated by the biplane Simpson method according to current recommendations [4], and the cut-off value for study inclusion was <40%. The left atrium (LA) was calculated using Simpson’s modified rule and indexed to the body surface area (BSA) [4]. The valvular function was assessed with 2D, color, pulsed, and continuous wave Doppler echocardiography [12].

#### 2.2.2. Two-Dimensional Speckle-Tracking Echocardiography

The 2D-STE-evaluated myocardial function with layer-specific myocardial deformation using the quantitative analysis (Q analysis) function allowed for comprehensive automated layer-specific analysis: endocardium, mid-myocardial, and epicardium. Aortic valve opening, aortic valve closure, mitral valve opening, and mitral valve closure were evaluated by pulsed wave Doppler obtained from the LV outflow tract and mitral diastolic flow, respectively. End systolic frame was defined by the closure of Ao valve in apical three-chamber view. The endocardial border was manually traced during the end-systole. The region of interest (ROI) was adjusted to encompass the entire thickness of the myocardium. If the limit of the endocardium seemed inaccurate, the region of interest (ROI) will be defined again, correcting the endocardial border, readjusting the ROI, or selecting a new ROI. Finally, the observer checks and validates the procedure. The adequacy of the tracking was verified visually, with manual adjustment of both the endocardial and epicardial border in cases of suboptimal tracing [6,7]; GCS evaluation at the level of MV, PM, or apical region of the LV resulted from manual tracing of the endocardial border in the end-systolic frame, defined by the Ao valve closure. The ROI was also adjusted to include the entire myocardium. The semiautomated strain algorithm traced the myocardial motion in the short-axis view. As mentioned above, the observer will also check and readjust, if necessary. The software automatically measured longitudinal mid-wall strain (GLS), longitudinal endocardial strain (GLSend), and longitudinal epicardial strain (GLSepi); circumferential strain at the MV level: mid-wall (CSMV), endocardial CSMVend, and epicardial CSMVepi, at the PM level: mid-wall CSPM, endocardial CSPMend, epicardial CSPMepi; and at the apical level: mid-wall CSAP, endocardial CSAPend, epicardial

**Figure 1:**
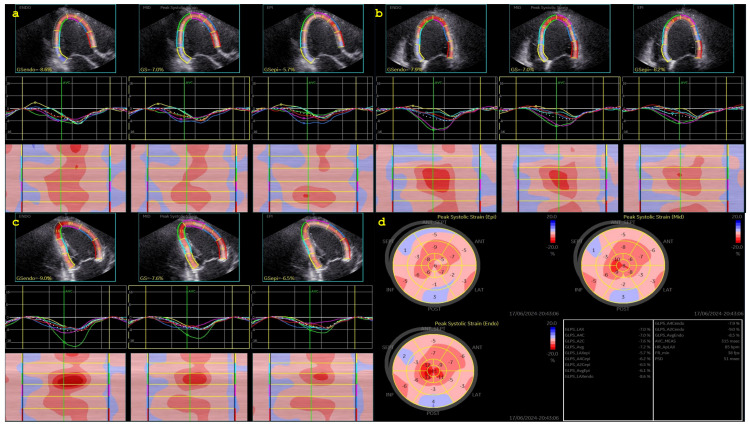
Layer-specific longitudinal strain analysis. (**a**) Apical 3-chamber view. (**b**) Apical 4-chamber view. (**c**) Apical three-chamber view. (**d**) Bulls-eye display of layers of strain.

**Figure 2:**
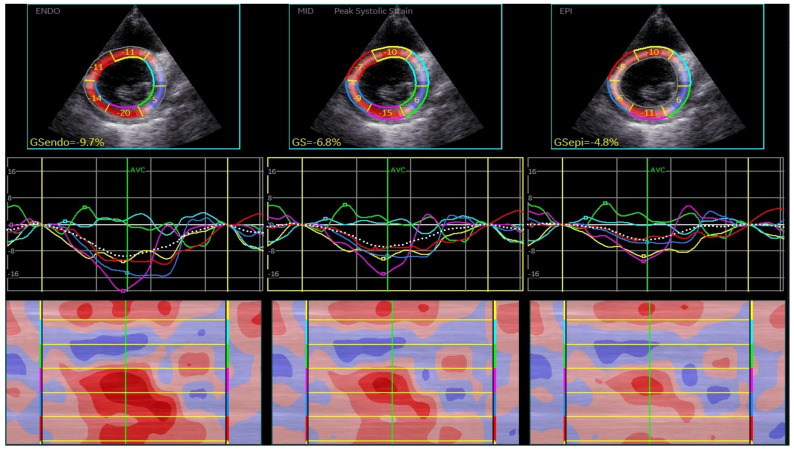
Layer-specific circumferential strain analysis at the level of mitral valve.

**Figure 3:**
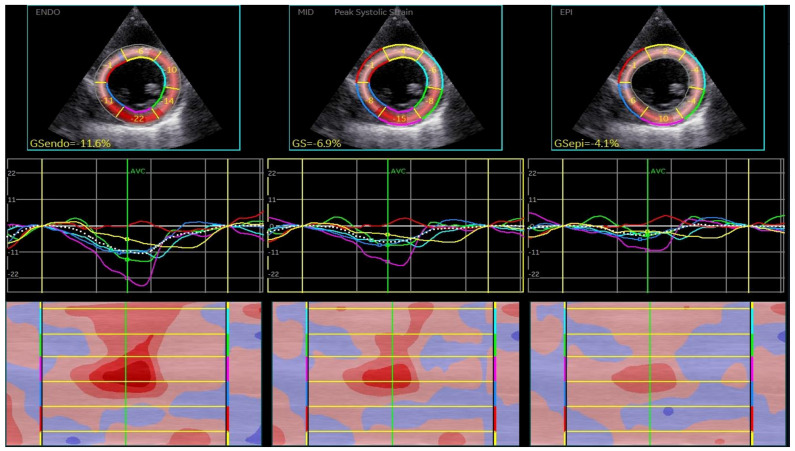
Layer-specific circumferential strain analysis at the level of papillary muscle.

**Figure 4:**
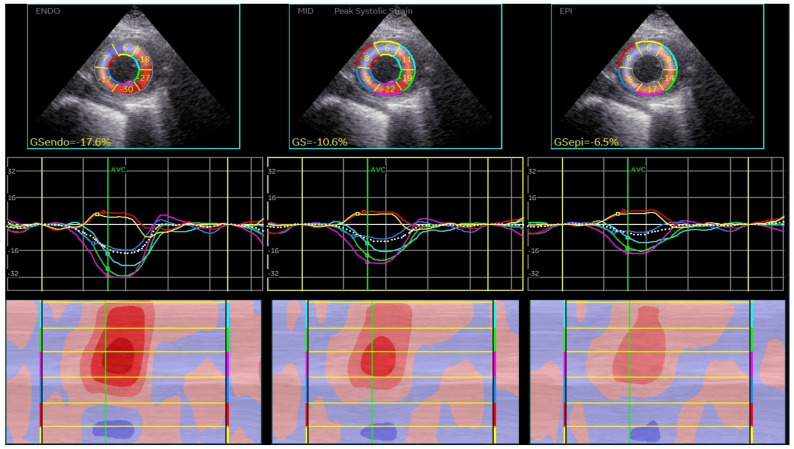
Layer-specific circumferential strain analysis at the apical level.

CSAPepi. Peak systolic longitudinal and circumferential strain from 3 layers assessed in all 18 segments of the LV were averaged to obtain longitudinal strain (Figure 1, BE layers) and circumferential strain (GCS) for each level (Figure 2, Figure 3 and Figure 4).

We also calculated the difference between endocardial and epicardial strain both in longitudinal (GLSend-GLSepi) and in the circumferential direction at the base (CSMVend-CSMVepi), mid-level (CSPMend-CSPMepi), and apical (CSAPend-CSAPepi) level of the LV. A visible endocardial border throughout the cardiac cycle and the correct position of the apex and mitral annulus were essential for GLS in three apical views and short-axis views at the base, mid-level, and apical segments of the LV, excluding the pericardium and papillary muscles because these structures could impact GLS values [13,14,15]. The subjects with unsatisfactory tracking were excluded from the study. Patients were followed for two years for all-cause mortality, defined as cardiac and non-cardiac mortality.

Statistical analysis was performed with IBM SPSS Statistics version 26.0.1.1. Baseline characteristics and echocardiographic parameters were defined as mean ± standard deviation (SD) for continuous variables and absolute number (n) or percentage (%) for categorical variables. The normal or skewed distribution of the variables was checked using the Kolmogorov–Smirnov test. To compare data between the two pre-defined groups, we used an unpaired *t*-test. Binary logistic regression (simple and multivariate) analysis was used to evaluate which clinical and conventional echocardiographic parameters were associated with two years of mortality, calculating odds ratios (ORs) with a 95% confidence interval (CI), respectively. Standard echocardiographic parameters qualified as independent prognostic parameters of two-year mortality in univariate analysis were included in a multivariate regression analysis. Similarly, all STE parameters were verified by logistic univariate regression for two-year mortality prediction, and ROC analysis was performed for each. Then, a model was created using multivariate stepwise regression to find STE layer-strain parameters qualified to have an incremental value of two-year mortality prediction in DCM patients with advanced stages of HF. Based on the receiver-operating characteristic (ROC) curve, the best cut-off value was considered the optimal point with the highest sum of sensitivity and specificity for predicting cardiac mortality. Significance was defined as a two-tailed probability level of *p* < 0.05 for all tests.

A single experienced sonographer performed the acquisition and postprocessing of echocardiographic images. Intra-observer reliability was assessed by re-analyzing the images of 10 randomly selected patients 30 days after the first analysis by the same operator. Intra-observer variability of multilayer strain was assessed by calculating the strain components’ intra-class correlation coefficient (ICC) and 95% confidence intervals (CIs).

## 3. Results

### 3.1. The General Characteristics of the Study Population Are Summarized in Table 1

The ischemic group included 51 patients (52.57%) with a mean age of 62.74. The patients in the nonischemic group were statistically significantly younger than patients in the ischemic group (55.73 +/− 11.68 years vs. 62.25 +/− 9.94 years, *p* = 0.0036). Of the subjects, 64.94% were men (60.78% in the ischemic group and 69.56% in the nonischemic group). The two groups had similar BSA, blood pressure, and heart rate (Table 1).

Cardiovascular risk factors—arterial hypertension, diabetes mellitus (DM), and dyslipidemia—were more prevalent in the ischemic group, and a higher proportion of patients with chronic kidney disease (CKD) was found in the ischemic group compared with the nonischemic group (Table 1). The prevalence of atrial fibrillation (AF) was 15.27% in the ischemic group and 11.76% in the nonischemic group. The proportion of non-survivors at two-year follow-up was higher in the nonischemic group, 26.08%, vs. the ischemic group, 17.64% (*p* = 0.32) (Table 1). The LV end-diastolic volume (LVEDV) and LV end-systolic volume (LVESV) were higher in patients with NIDCM, and LVEF was lower (Table 2). Left atrium volume indexed (LAVi) was more dilated in the case of ischemic etiology compared with nonischemic etiology. No statistically significant differences were found in classical echocardiographic parameters between the two groups (Table 2).

The percentage of patients with MR ≥ mild and TR ≥ was higher in the NI group, as well as tenting area (TA), linked to mitral annulus remodeling (Table 2).

### 3.2. 2D Speckle-Tracking Echocardiography Results

GLS in the nonischemic group was lower compared with the ischemic group, −7.22 vs. −7.84 (*p* = 0.2851), as well as GLSend, −8.22 vs. −9.15 (*p* = 0.1471), respectively. The gradient between endocardial and epicardial longitudinal strain was higher in IDCM patients, −2.43 vs. −2.41 (*p* = 0.9373) (Table 3).

The same profile was found regarding the CS at the MV level—CSMV −7.74, CSMVend −5.58, and CSMVend-CSMVepi −2.43 in ischemic vs. −7.22 (*p* = 0.3682), −10.16 (*p* = 0.545), and −5.59 (0.7152) in the nonischemic group; at the PM level—CSPM −8.16, CSPMend −11.57, and CSPMend-CSPMepi −6.75 in IDCM vs. −7.19 (*p* = 0.1028), −10.5 (0.1834), and −5.78 (*p* = 0.1406) in NIDCM; and at the apical LV level—CSAP −10.19, CSAPend −13.77, CSAPend-CSAPepi −6.87 in ischemic vs. −8.65 (*p* = 0.0249), −11.67 (0.0369), and −5.17 (*p* = 0.0163) in nonischemic subjects (Table 3).

Logistic univariate analysis identified BSA, male gender, hypertension, CKD, LVEDV, LVESV, LVEF, and LAVi as mortality predictors at the two-year follow-up (Table 4). Hypertension (OR 5.9, 95% CI 1.3035 to 26.7044, *p* = 0.0212) and LAVi (OR 1.0683, 95% CI 0.5616 to 1.1236, *p* = 0.038) were independent predictors of two-year mortality in patients with DCM and advanced stages of HF using multivariate analysis (Table 5).

**Figure 5:**
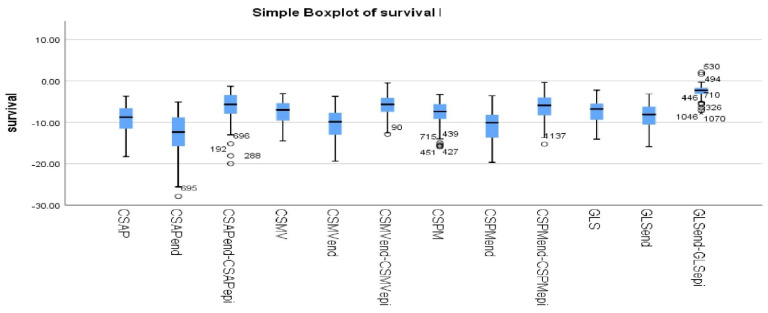
**Boxplots of strain parameters in survivors vs. non-survivors.** GLS—mid-layer longitudinal strain; GLSendendocardium longitudinal strain; GLSend-GLSepi—the gradient between endocardium longitudinal strain and epicardium longitudinal strain; CSMV—mid-layer circumferential strain at the level of the mitral valve; CSMVend—endocardium circumferential strain at the level of the mitral valve; CSMVend-CSMVepi—the gradient between endocardium circumferential strain and epicardium circumferential strain at the level of the mitral valve; CSPM—mid-layer circumferential strain at the level of the papillary muscles; CSPMend—endocardium circumferential strain at the level of the papillary muscles; CSPMend-CSPMepi—the gradient between endocardium circumferential strain and epicardium circumferential strain at the level of the papillary muscles; CSAP—mid-layer circumferential strain at the apical level; CSAPend—endocardium circumferential strain at the apical level; CSAPend-CSAPepi—the gradient between endocardium circumferential strain and epicardium circumferential strain at the apical level.

LV GLS and CS were more preserved in survivors than non-survivors in all layers. We found statistically significant differences between survivors and non-survivors in GLS, −8 vs. −6.2 (*p* = 0.0046); GLSend, −9.24 vs. −6.98; CSPMend, −7.99 vs. −6.64 (*p* = 0.0613); and CSAPend, −9.78 vs. −7.97 (*p* = 0.0477). Further, regarding the gradient between endocardial and epicardial strains, we obtained the following: GLSend-GLSepi, −2.705 vs. −1.86 (*p* = 0.0107); CSPMend-CSPMepi, −6.72 vs. −4.73 (*p* = 0.0116); CSAPend-CSAPepi, −6.44 vs. −4.71 (*p* = 0.0448) (Table 6). The differences in strain parameters between survivors and non-survivors are illustrated in Figure 5.

STE parameters that qualified as two-year mortality predictors in univariate analysis were GLS (OR 1.3784, 95% CI 1.0853 to 1.7507, *p* = 0.0085), GLSend (OR 1.3375, 95% CI 1.0822 to 1.6531, *p* = 0.0071), GLSend-GLSepi (OR 1.73671, 95% CI 1.1032 to 2.7339, *p* = 0.0067), CSPMend (OR 1.2366, 95% CI 1.0592 to 1.4437, *p* = 0.0072), CSPMend-CSPMepi (OR 1.2585, 95% CI 1.0449 to 1.5157, *p* = 0.0154), CSAPend (OR1.1562, 95% CI 1.0236 to 1.3059, *p* = 0.0196), and CSAPend-CSAPepi (OR 1.2035, 95% CI 1.0013 to 1.4464, *p* = 0.0484) (Table 7).

To find the incremental value of layer-specific strain parameters over conventional clinical and echocardiographic parameters, we introduced the factors identified by univariate analysis into a multivariate stepwise regression. Only CSPMend qualified as a parameter with the incremental predictive value of two-year mortality in DCM patients in an advanced stage of HF (OR 1.2492, 95% CI 1.0482 to 1.4887, *p* = 0.0129), added to hypertension and LAVi (Table 8).

**Figure 6:**
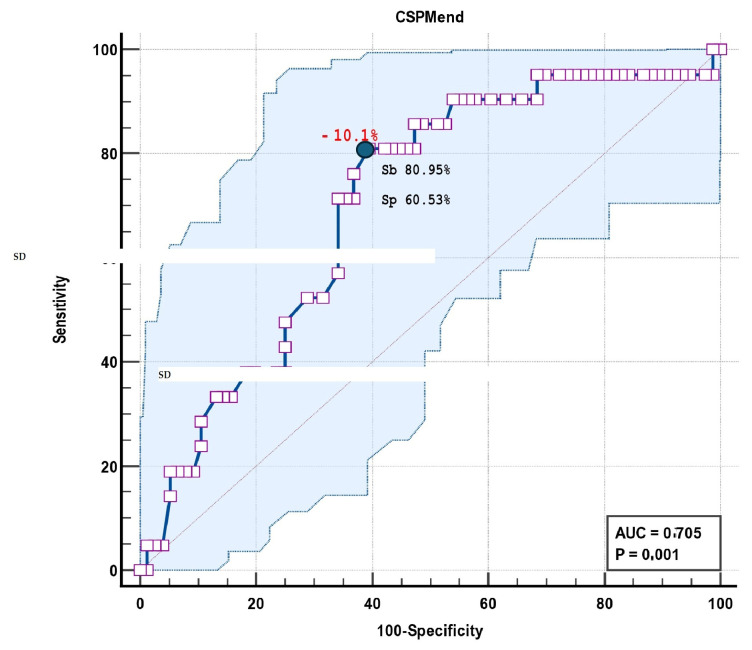
**ROC curve analysis** to identify sensitivity and specificity of CSPMend of −10.1% as an incremental factor to predict two-year mortality in patients with DCM and HF.

The model results were AUC 0.846, SE 0.0456, and 95% CI 0.759 to 0.912 (Table 9). ROC curve analysis identified a CSPMend of −10.1% as the cut-off value in differentiating the survivors and non-survivors, with a sensitivity of 80.95% and specificity of 60.53% (AUC 0.705, 95% CI 0.603 to 0.793) (Figure 6, Table 10).

Bootstrapping for the stepwise multivariate model, including CSPMend value validation, is provided in Table 11.

**Figure 7:**
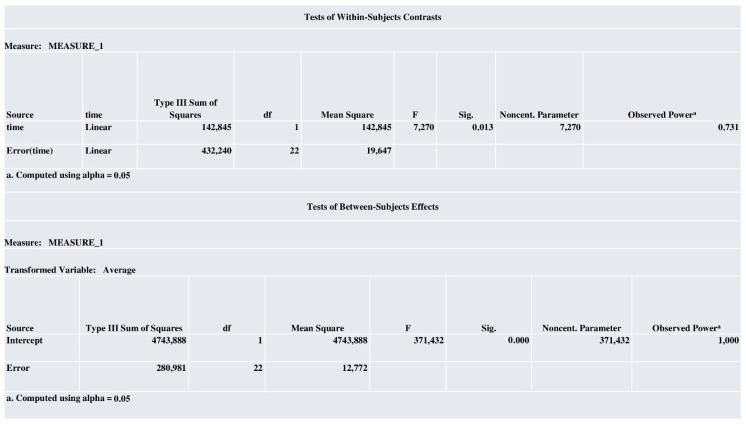
Power analysis report.

The power analysis report was relatively good, as revealed in Figure 7.

### 3.3. Reproducibility of the Measurements

The intra-observer reproducibility was excellent for GLS measurements. Variability was low for longitudinal strains. Circumferential strains presented higher variability at the basal level of the heart. A very good reproducibility was obtained for CSPMend, 0.938 (CI 0.771 to 0.984) (Table 11).

**Table 11:** Bootstrapping for the stepwise multivariate model, including CSPMend value validation.

Bootstrap for Coefficients							
Model		B	Bootstrap				
			Bias	Std. Error	Sig. (2-Tailed)	95% Confidence Interval	
						Lower	Upper
1	(Constant)	0.046	0.000	0.112	0.683	−0.163	0.275
	CSPMend	0.025	0.000	0.002	0.001	0.020	0.029
	LAVi	0.011	−7.062 × 10^−6^	0.002	0.001	0.007	0.014
	hypertension	−0.215	−0.002	0.033	0.001	−0.282	−0.151

## 4. Discussion

In the current study, we analyzed circumferential and longitudinal strain in patients with DCM and HF and performed layer-specific strain analyses. We evaluated the differences in layer-strain according to ischemic etiology, between two-year survivors and non-survivors, and the cut-off values that could predict mortality. The main findings of our study can be summarized as follows: (1) In all DCM patients, GLS and CS were decreased. (2) Patients with NIDCM presented lower layer-specific strain values than IDCM patients but without statistically significant differences. (3) At the two-year follow-up, non-survivors had more impaired LV, GLS, and CS at all layers (endomyocardial, mid-myocardial, and epicardial), suggesting more extensive scar tissue at baseline echocardiography when compared to survivors. (4) The multivariable analysis demonstrated that CSPMend could have incremental value for predicting two-year mortality in DCM patients with advanced stages of HF, after adjusting for hypertension and conventional echocardiographic variables such as LAVi. Finally, LV layer-specific strain analysis using 2DTTE could play a central role in evaluating LV systolic function in this group of patients (Table 12).

This is the first study to analyze the utility of layer-specific strain analysis in patients with DCM in an advanced stage of HF, according to etiology.

The patients in our study had similar baseline characteristics (BSA, heart rate, blood pressure). The prevalence of cardiovascular risk factors was higher in the ischemic group, although more severe echocardiographic parameters were found in patients with NIDCM. After evaluating the left heart chambers and MV, we found that patients with NIDCM, compared to those with IDCM, presented higher LV volumes and lower values of LVEF. More than mild MR was present in the NIDCM group, translating into higher tenting area (TA) values. The ischemic group displayed increased LAVi. All strain parameters, longitudinal and circumferential, global, and layer-specific, were more damaged in the nonischemic group.

LV geometry had a significant impact on GLS, but extensive studies involving all three layers of the LV, in the longitudinal and circumferential directions, are scars. Some research provided lower values of segmental strain but used a single vendor [16]. LVEF is a summation of the circumferential and longitudinal movement. Using EF as a single functional parameter cannot appropriately describe myocardial mechanics [17].

The left ventricular (LV) wall comprises three layers of fibers. The opposite orientation of the myocardial fibers in the subendocardial and subepicardial layers is essential for the equal redistribution of stress and strain in the heart [5,7]. The endocardial layer consists of longitudinally orientated fibers and is more load-dependent than the other layers. The mid-myocardial layer consists of circumferentially arranged fibers and occupies two-quarters of the thickness. The outer layer is the thinnest, in contact with the pericardium, and has the lowest longitudinal deformation values [18]. Myocardial deformation consists of three orthogonal components: longitudinal, radial, and circumferential. Myocardial fibers contribute differentially to longitudinal and circumferential shortening, depending on their angulation [5]. The fibers in the subendocardial layer contribute to longitudinal shortening. Oblique and circumferentially orientated fibers in the subepicardial layer contribute to circumferential shortening and rotation [19]. Assessment of layer strain parameters by STE allows for the separate interrogation of these different functional units.

Pedrizetti et al. showed that EF could be expressed as a function of GLS and GCS [20]. The most frequently used deformation component is LS [5]. For a given pre- and afterload, the shortening of myofibers is determined by their intrinsic contractility, which may be damaged by fibrosis and depositions. Long-lasting volume overload produces a vicious cycle of progressive LV chamber enlargement, increasing wall stress and contractile dysfunction with reduced strain values [21]. In normal conditions, the outer LV layer mainly causes CS and contributes 17% to the total LV wall thickening. The inner third of the LV wall contributes 58% to the thickening, resulting in a gradient between the endocardium and epicardium [7,19,22,23,24,25]. Increased end-diastolic wall stress toward the endocardium contributes to the endocardium–epicardium gradient. The endocardial fibers are stretched longer than the epicardial fibers during end-diastole, increasing fiber shortening in the endocardial layer during systole [26]. Coronary perfusion and metabolism differences between the endocardial and epicardial layers could also explain the transmural strain gradient [27]. Subendocardial layer damage will predominantly influence longitudinal shortening, and all myocardial layer damage will influence longitudinal shortening and circumferential strain, twist, and torsion. The strain parameters correlated with EF and fibrosis [28,29,30].

GLS has incremental diagnostic and prognostic value in many cardiac diseases [31]. Longitudinal deformation is preserved in the LV apical segments (apical sparing), suggesting amyloidosis in patients with LV hypertrophy [31]. Early septal stretch combined with late lateral wall contraction predicted the response to the therapy in subjects referred to cardiac resynchronization therapy [32]. Regional myocardial dysfunctions assessed with STE correlated with fibrosis identified with cardiac magnetic resonance (CMR) and had prognostic value in acute myocarditis [33,34].

Multi-layer STE allows analysis of endomyocardial, mid-myocardial, and epicardial functions in different diseases [7,35,36]. It is still unknown which layer has the best prognostic value in various cardiac diseases. Hamada et al. evaluated LV strain in patients with chronic ischemic cardiomyopathy. GLSend was associated with adverse cardiac events (readmission, worsening of HF, ventricular arrhythmias, and all-cause mortality) independent of LVEF and transmural scar identified by CMRI [37]. In the study of Skaarup et al. [38], evaluating patients after STEMI, non-STEMI, and unstable angina, LV GLS measured at all layers was associated with HF and cardiovascular death. In ischemic heart disease, only GLS and GLSepi seemed to be more decisive prognostic factors for adverse outcomes after adjusting for clinical and echocardiographic parameters, and only LV GLSepi was independently associated with cardiac death. The authors also suggested that a layer-specific analysis could help discriminate between transmural and subendocardial infarction [38].

The mid-myocardium and epicardial layers mainly contribute to radial and circumferential LV systolic function. A preserved mid myocardium and epicardial layer function prevents further LV deterioration, reflecting the extent of affected LV myocardial tissue after an ischemic event [39,40]. Abou et al. also reported that LV GLS measured at the mid-myocardium and the epicardium reflected the presence of transmural scar associated with increased mortality in STEMI patients with mildly reduced or preserved LVEF [41]. In patients with acute coronary ischemia, the studies identified immediate changes in regional myocardial deformation [42]. GLSend was superior to GLSepi in diagnosing coronary artery disease [43] and outcomes [37,44,45]. The study of Dahlslett et al. showed that endocardial longitudinal and circumferential strains were better at identifying patients with significant coronary artery disease [46]. Ischemia damages the subendocardial layer of the myocardium, while the patients with transmural myocardial infarction present fibrotic replacement of the myocardium, with damage to all layers [47,48]. Decreased strain at the epicardium level discriminated between non-transmural and transmural scar areas [49]. GLSepi provided incremental prognostic value in acute coronary syndrome [38], hypertension [50], myocarditis [51], and HCM [52].

Despite the treatment progress, HF with reduced EF is one of the most important causes of hospitalization and mortality [53]. LVEF is a prognosis parameter for many diseases, but it does not reflect the LV mechanics of myocardial fibers [54]. LVEF is a significant predictor of survival in patients with DCM [55]. The most recent guidelines recommend LVEF assessment as the primary measurement of LV systolic function in patients with DCM [4]. However, LV GLS is superior to LVEF in reproducibility and prediction of complex events such as all-cause and cardiovascular mortality [29,56].

Each myocardial layer is differently affected in HF, and this would also affect long-term prognosis. CMR studies have suggested that NIDCM affects all layers of the myocardium similarly, whereas in IDCM patients, dysfunctional fibers are located primarily in the subendocardium. Etiologies affecting primarily subendocardial fibers will predominantly influence longitudinal shortening, and etiologies affecting all myocardial layers will influence both longitudinal and circumferential strain. In the early phase of HF, GCS often compensates for an early loss of longitudinal function. The damage of both subendocardial and subepicardial layers might cause more advanced limitation of functional capacity, affecting the long-term prognosis [57]. In advanced stages of the disease, with extensive fibrosis, GLS of IDCM patients was not different from that of NICM patients, but some authors reported that GCS can differentiate the two entities [58,59].

In our study, LVEF was smaller in NIDCM subjects but without statistically significant differences in the two groups (Table 2). GLS, CS, and layer-specific strain analysis provided smaller values in NIDCM patients compared with IDCM patients, especially in the apical regions of the LV (Table 3). These findings might suggest more extensive fibrosis and advanced remodeling in patients with nonischemic etiology for comparable LVEF.

The scar pattern is subendocardial or transmural in patients with coronary artery disease, and endocardial strain analysis has been shown to have superior prognostic performance [44]. The scar is located in the mid-wall or subepicardium in patients with nonischemic cardiomyopathies [37].

Some studies reported similarities between GLS-specific strain analysis by CMR and STE [58,59], identifying differences between GLSend and GLSepi, significantly reducing from HFpEF and HFmrEF to HFrEF [59]. Xu et al. identified GLS, GLSend, GLSepi, and LVEF as significantly predictive of outcomes in univariate analysis, but only GLS remained independently associated with clinical outcomes [59]. Our study parameters predictive of two-year mortality in univariate analysis were GLS, GLSend, GLSepi, CSPMend, CSPMend-CSPMepi, CSAPend, and CSAPend-CSAPepi. However, only CSPMend qualified in multivariate analysis as an incremental parameter to clinical factors such as hypertension and standard echocardiographic factors such as LAVi in predicting survival in patients with DCM and advanced stages of HF. Previous CMR studies also showed that GLSend was superior to GLSepi [60].

As in Adamu et al.’s study, we found that CS was reduced in all patients in all layers, with a reduced gradient between endocardial and epicardial layers [61]. Similar to our study, Zhang et al. reported advanced damage of all myocardial strains in patients with NSTE-ACS due to complex lesions. They also noted that the most affected was the endocardium, with a decrease in the gradient between the endocardium and epicardium [62]. The more advanced CAD, the lower the absolute differences between endocardial and epicardial GLS and CS [62]. This conclusion might be in accordance with our results with a decreased value of strain in all layers. In patients with DCM and advanced stages of HF, the gradient between endocardial and epicardial strain was decreased, without significant differences between ischemic and nonischemic etiology of the disease. The values were lower in patients with NIDCM, sustaining a more extensive level of fibrosis. The endocardial GLS and CS seem to have a better diagnostic accuracy than GCS, CS, mid-myocardial and epicardial GLS, and CS [62].

Studies conducted in patients with coronary artery disease showed that endocardial layer longitudinal function is impaired at the early phase of ischemia. At the same time, the mid-layer and epicardium could be involved in a compensation function [63,64] resulting in reduced GLS, layers LS, normal or increased CS, and layers CS. In the advanced stage of the disease, ischemia extends from the endocardium to the epicardium [48], being transmural, decreasing all layers’ functions, but the endocardium is the most damaged. Another aspect that must be considered is that each myocardial layer deformation is not independent. It is the sum of an active contraction within the layer and the passive influence of the adjacent layer [43]. These studies concluded that endocardial longitudinal and circumferential strains decreased more obviously than GLS and LLS in ischemic heart disease.

With increasing numbers of transmural necrotic segments, in advanced stages of the disease, IDCM begins to resemble NIDCM with an increasing region of the miocardialcardial layers being dysfunctional. Consequently, strain parameters have the same features and can be measured in both groups [65]. These findings are in accordance with our study. We did not find statistically significant differences in the LSS analysis between the two entities, nor in the gradients between endocardial and epicardial layers in the longitudinal and circumferential directions. These results confirm the CMR studies that showed that, in advanced stages of the NIDCM and IDCM, fibrosis is the pathological mechanism leading to ventricular dysfunction, stiffness, cardiac remodeling, and HF. If GLS is reduced in the early stages of cardiomyopathy, a lowering of CS is a phenomenon in advanced disease [65]. In our study, evaluating patients with advanced HF and reduced LVEF, both longitudinal and circumferential strain at the level of MV, PM, and AP were blunted. The strain layers were reduced at the level of the endocardium, mid-myocardium, and the difference between the endocardium and epicardium for longitudinal and circumferential strain. Compared with IDCM, patients with NIDCM presented more decreased strain in all longitudinal and circumferential layers but without statistical significance between the groups, except the apical zones of the LV, sustaining similarity between the two groups in this stage of the disease.

The IDCM group in the current study was slightly older than the NIDCM group. Despite this difference, the strain was higher in subjects with ischemic etiology. This was not a limitation because of the relatively small decline in strains beyond 50 years of age [66]. In the study of Nagata Y et al., including healthy volunteers, the layer-specific global strain parameters showed no age dependency but had gender dependency, except for endocardial CS. A subgroup analysis of this study revealed that apical endocardial LS was preserved even in elderly subjects [25]. Age was not an independent predictor of outcome in our study.

This is the first echocardiographic STE study demonstrating differences between layer-specific strain in patients with ischemic and nonischemic etiology of DCM despite phenotypically identical aspects. Different distributions of fibrosis between layers could explain these features. Vietheer Jet obtained similar results using CMR strain analysis and found the same similitude between IDCM and NIDCM strains. They explained this feature by increasing the number of transmural segments in ICM that begin to resemble DCM as an increasing portion of the epicardial layers is dysfunctional [67]. Layer-specific strain analysis can identify differences in myocardial mechanics between ICM and DCM, even if 2D echocardiographic features are similar in advanced stages of HF. Layer strain measured on the endocardium, mid-level, or epicardium is not equivalent to identifying dysfunction or outcomes prediction in HF.

Similar to previous CMR research that concluded CS is more severely damaged in an advanced stage of the disease compared with GLS, our study revealed CSPMend becoming important in advanced stages of HF. The cut-off value of −10.1% had 80.95% sensitivity, but with only 60.53% specificity in predicting the probability of two-year mortality in this group of patients, after adjusting for hypertension and conventional echocardiographic variables such as LAVi.

The deformation of each myocardial segment is not independent. Contraction of the viable myocardium influences the deformation of the adjacent nonviable myocardium by traction. The nonviable myocardium may also negatively affect the contraction of the adjacent viable myocardium. Each layer’s deformation results from the active contraction within that layer and the passive traction of the adjacent myocardium [44,46]. LSS analysis has good intra- and inter-observer reproducibility but poor inter-vendor reproducibility [68].

## 5. Conclusions

Layer strain measured on the endocardium, mid-level, or epicardium is not equivalent to identifying dysfunction or outcomes prediction in patients with DCM. There are some differences in the advanced stages of HF disease despite similar phenotypes due to extensive fibrosis. Nonischemic etiology is linked to lower strain values at the level of all layers. CSPMend was an incremental prognostic factor, after adjusting for hypertension and conventional echocardiographic variables such as LAVi that could predict the probability of mortality in this group of patients. Further studies with a larger number of patients are necessary to confirm these results.

## 6. Study Limitation

Firstly, this was a single-center study.

Secondly, it was a study with a relatively small number of patients and should be verified on a larger cohort.

Most subjects were male, and there might be differences between males and females regarding layer-specific strain analysis in DCM patients.

The study did not focus on DCM etiology except for coronary disease.

Thirdly, the analysis was performed with a single vendor-dependent software (Echo Pac v204), so the results are vendor-specific.

Fourth, the image acquisition and post-processing were performed by a single sonographer.

Fifth, this was a retrospective analysis and may call for some selection bias.

## Figures and Tables

**Table 1:** **Baseline characteristics in patients with DCM.** Data are presented as number (n), percentage (%), and mean ± standard deviation. Abbreviations: DCM—dilated cardiomyopathy; IDCM—ischemic dilated cardiomyopathy; NIDCM—nonischemic dilated cardiomyopathy; BSA—body surface area; DM—diabetes mellitus; CKD—chronic kidney disease. *p*-value was calculated using the *t*-test for comparison of means and *t*-test for comparison of proportions, AF—atrial fibrillation.

Parameter	IDCM (51) (52.57%)	NIDCM (46) (47.24%)	*p*
Age (years)	62.25 +/− 9.94	55.73 +/− 11.68	0.0036
Males (%)	60.78	69.56	0.37
BSA (m^2^)	1.77 (0.16)	1.78 (0.17)	0.76
Hypertension (%)	74.5	54.34	0.039
DM (%)	52.94	45.65	0.47
Dyslipidemia (%)	64.7	63.07	0.86
Obesity (%)	41.17	32.6	0.38
CKD (%)	39.21	21.73	0.063
AF (%)	13.72	16.66	0.69
Deaths (%)	17.64	26.08	0.32

**Table 2:** **Standard echocardiographic parameter measurements in patients with DCM.** Data are presented as mean ± standard deviation. Abbreviations: DCM—dilated cardiomyopathy; IDCM—ischemic dilated cardiomyopathy; NIDCM—nonischemic dilated cardiomyopathy; LVEDV—left ventricular end-diastolic volume; LVESV—left ventricular end-systolic volume; LVEF—left ventricular ejection fraction; LAVi—left atrium volume indexed; MR—mitral regurgitation; TR—tricuspid regurgitation; TA—tenting area. *p*-value is calculated using the *t*-test for comparison of means and *t*-test for comparison of proportions.

Parameter	IDCM (51) (52.57%)	NIDCM (46) (47.24%)	*p*
LVESV (mL)	192.19 (64.09)	203.97 (81.26)	0.43
LVEDV (mL)	141.11 (55.27)	152.06 (70.75)	0.39
LVEF (%)	27.05 (7.93)	26.58 (7.48)	0.35
LAVi (mL/m^2^)	55.23 (11.23)	54.16 (14.69)	0.68
MR ≥ mild (%)	33.33	41.3	0.42
TR > mild (%)	25.49	39.13	0.68
TA	4.29 (0.7)	4.31 (0.98)	0.9

**Table 3:** **Layer-specific strain analysis in patients with DCM.** Data are presented as mean ± standard deviation. Abbreviations: DCM—dilated cardiomyopathy; IDCM—ischemic dilated cardiomyopathy; NIDCM—nonischemic dilated cardiomyopathy; GLS—mid-layer longitudinal strain; GLSend—endocardium longitudinal strain; GLSend-GLSepi—the gradient between endocardium longitudinal strain and epicardium longitudinal strain; CSMV—mid-layer circumferential strain at the level of the mitral valve; CSMVend—endocardium circumferential strain at the level of the mitral valve; CSMVSend-CSMVSepi—the gradient between endocardium circumferential strain and epicardium circumferential strain at the level of the mitral valve; CSPM—mid-layer circumferential strain at the level of the papillary muscles; CSPMend—endocardium circumferential strain at the level of the papillary muscles; CSPMend-CSPMepi—the gradient between endocardium circumferential strain and epicardium circumferential strain at the level of the papillary muscles; CSAP—mid-layer circumferential strain at the apical level; CSAPend—endocardium circumferential strain at the apical level; CSAPend-CSAPepi—the gradient between endocardium circumferential strain and epicardium circumferential strain at the apical level. *p*-value was calculated using the *t*-test for comparison of means.

Parameter	IDCM MV	SD	NIDCM MV	SD	Diff	SE	95% CI DF −95	t	*p*
GLS	−7.84	2.92	−7.22	2.74	0.620	0.577	−0.5249 to 1.7649	1.075	0.2851
GLSend	−9.15	3.24	−8.22	3	0.930	0.636	−0.3330 to 2.1930	1.462	0.1471
GLSend-GLSepi	−2.43	1.48	−2.41	0.92	0.020	0.253	−0.4832 to 0.5232	0.079	0.9373
CSMV	−7.74	2.88	−7.22	2.77	0.520	0.575	−0.6218 to 1.6618	0.904	0.3682
CSMVend	−10.6	3.67	−10.16	3.44	0.440	0.724	−0.9983 to 1.8783	0.607	0.5451
CSMVSend-CSMVSepi	−5.8	2.76	−5.59	2.89	0.210	0.574	−0.9293 to 1.3493	0.366	0.7152
CSPM	−8.16	3.07	−7.19	2.69	0.970	0.589	−0.1991 to 2.1391	1.647	0.1028
CSPMend	−11.57	3.96	−10.5	3.88	1.070	0.799	−0.5153 to 2.6553	1.340	0.1834
CSPMend-CSPMepi	−6.75	3.02	−5.78	3.4	0.970	0.653	−0.3261 to 2.2661	1.486	0.1406
CSAP	−10.19	3.65	−8.65	3.62	1.690	0.741	0.2183 to 3.1617	2.280	0.0249
CSAPend	−13.77	4.67	−11.67	5.1	2.100	0.992	0.1307 to 4.0693	2.117	0.0369
CSAPend-CSAPepi	−6.87	3.71	−5.17	3.06	1.700	0.695	0.3204 to 3.0796	2.446	0.0163

**Table 4:** Logistic univariate analysis for clinical and conventional echocardiographic parameters in predicting cardiac two-year mortality in DCM patients. Data are presented as mean ± standard deviation. Abbreviations: DCM—dilated cardiomyopathy; SE—standard error; OR—odds ratio; CI—confidence interval; AUC—area under the curve; BSA—body surface area; DM—diabetes mellitus; CKD—chronic kidney disease; AF—atrial fibrillation; LVEDV—left ventricular end-diastolic volume; LVESV—left ventricular end-systolic volume; LVEF—left ventricular ejection fraction; LAVi-left atrium volume indexed; MR—mitral regurgitation; TA—tenting area.

Parameter	Coefficient	SE	OR	95% CI	AUC	*p*
Age	−0.0055909	0.022025	0.9944	0.9524 to 1.0383	0.504	0.8
BSA	4.67658	1.65489	107.4017	4.1912 to 2752.2135	0.717	0.0047
Male	−1.41908	0.66586	0.2419	0.0656 to 0.8923	0.633	0.0331
Hypertension	1.18562	0.50835	3.2727	1.2084 to 8.8639	0.641	0.0197
DM	−0.85137	0.51696	0.4268	0.1550 to 1.1757	0.603	0.0996
Dyslipidemia	0.053489	0.50840	1.0549	0.3895 to 2.8575	0.506	0.9162
CKD	1.45775	0.51695	4.2963	1.5597 to 11.8341	0.667	0.0048
AF	0.095310	0.70996	1.1000	0.2736 to 4.4230	0.506	0.8932
LVEDV	0.0073219	0.0033724	1.0073	1.0007 to 1.0140	0.638	0.0299
LVESV	0.010617	0.0042349	1.0107	1.0023 to 1.0191	0.704	0.0122
LVEF	−0.12205	0.038815	0.8851	0.8203 to 0.9551	0.740	0.0017
LAVi	0.073302	0.021835	1.0761	1.0310 to 1.1231	0.733	0.0002
MR	0.55862	0.49937	1.7483	0.6569 to 4.6524	0.567	0.2633
TA	0.53870	0.28886	1.7138	0.9729 to 3.0188	0.604	0.0622

**Table 5:** Multivariate analysis model identifying clinical and classical echocardiographic parameters predictors of two-year mortality in DCM patients. Data are presented as mean ± standard deviation. Abbreviations: DCM—dilated cardiomyopathy; SE—standard error; OR—odds ratio; CI—confidence interval; BSA—body surface area; CKD—chronic kidney disease; LVEDV—left ventricular end-diastolic volume; LVESV—left ventricular end-systolic volume; LVEF—left ventricular ejection fraction; LAVi—left atrium volume indexed.

Parameter	Coefficient	SE	OR	95%CI	*p*
BSA	4.94503	3.48548	140.4758	0.1516 to 130.168	0.1560
Males	0.45761	0.91754	1.5803	0.2616 to 9.5446	0.6180
Hypertension = 0	1.77494	0.77035	5.9000	1.3035 to 26.7044	0.0212
CKD = 1	1.40238	0.75463	4.0648	0.9262 to 17.8402	0.0631
LVEDV	0.041878	0.068994	1.0428	0.9109 to 1.1938	0.5439
LVESV	−0.058942	0.084732	0.9428	0.7985 to 1.1131	0.4867
LVEF	−0.23021	0.17691	0.7944	0.5616 to 1.1236	0.1932
LAVi	0.066052	0.031852	1.0683	0.5616 to 1.1236	0.0381

**Table 6:** **Layer-specific strain mean value at discharge in non-survivors vs. survivors of DCM during two-year follow-up.** Data are presented as mean ± standard deviation. Abbreviations: DCM—dilated cardiomyopathy; SE—standard error; CI—confidence interval; DF—degree of freedom; GLS—mid-layer longitudinal strain; GLSend—endocardium longitudinal strain; GLSend-GLSepi—the gradient between endocardium longitudinal strain and epicardium longitudinal strain; CSMV—mid-layer circumferential strain at the level of the mitral valve; CSMVend—endocardium circumferential strain at the level of the mitral valve; CSMVSend-CSMVSepi—the gradient between endocardium circumferential strain and epicardium circumferential strain at the level of the mitral valve; CSPM—mid-layer circumferential strain at the level of the papillary muscles; CSPMend—endocardium circumferential strain at the level of the papillary muscles; CSPMend-CSPMepi—the gradient between endocardium circumferential strain and epicardium circumferential strain at the level of the papillary muscles; CSAP—mid-layer circumferential strain at the apical level; CSAPend—endocardium circumferential strain at the apical level; CSAPend-CSAPepi—the gradient between endocardium circumferential strain and epicardium circumferential strain at the apical level. *p*-value was calculated using the *t*-test for comparison of means, SD—standard deviation, MV—mean value.

Parameter	DCM Nonsurvivors (21) MV	SD	DCM Survivors (76) MV	SD	Diff	SE	CI DF 95	t	*P*
GLS	−6.02	1.52	−8	3.01	−1.980	0.682	−3.3333 to −0.6267	−2.973	0.0046
GLSend	−6.98	1.89	−9.24	3.33	−2.260	0.760	−3.7690 to −0.7510	−2.973	0.0037
GLSend-GLSepi	−1.86	0.74	−2.705		−0.900	0.345	−1.5858 to −0.2142	−2.605	0.0107
CSMV	−7.07	3.26	−7.56	1.53	−0.290	0.700	−1.6806 to 1.1006	0.414	0.6798
CSMVend	−9.58	4.19	−10.62	3.35	−1.040	0.874	−2.7742 to 0.6942	−1.191	0.2368
CSMVend-CSMVepi	−4.66	2.72	−5.98	2.77	−1.320	0.682	−2.6745 to 0.0345	−1.935	0.0560
CSPM	−6.64	2.62	−7.99	2.96	−1.350	0.713	−2.7653 to 0.0653	−1.894	0.0613
CSPMend	−8.92	3.28	−11.66	3.92	−2.740	0.935	−4.5970 to −0.8830	−2.929	0.0043
CSPMemd-CSPMepi	−4.73	2.62	−6.72	3.26	−1.990	0.773	−3.5249 to −0.4551	−2.574	0.0116
CSAP	−7.97	2.73	−9.78	3.87	−1.810	0.902	−3.6011 to −0.0189	−2.006	0.0477
CSAPend	−10.48	3.47	−13.41	5.14	−2.930	1.192	−5.2971 to −0.5629	−2.457	0.0158
CSAPend-CSAPepi	−4.71	2.61	−6.44	3.64	−1.730	0.851	−3.4187 to −0.0413	−2.034	0.0448

**Table 7:** **Logistic univariate analysis model in identifying STE parameter predictors of two-year mortality in DCM patients.** Data are presented as mean ± standard deviation. Abbreviations: DCM—dilated cardiomyopathy; SE—standard error; OR—odds ratio; CI—confidence interval; AUC—area under the curve; GLS—mid-layer longitudinal strain; GLSend-endocardium longitudinal strain; GLSend-GLSepi—the gradient between endocardium longitudinal strain and epicardium longitudinal strain; CSMV—mid-layer circumferential strain at the level of the mitral valve; CSMVend—endocardium circumferential strain at the level of the mitral valve; CSMVend-CSMVepi—the gradient between endocardium circumferential strain and epicardium circumferential strain at the level of the mitral valve; CSPM—mid-layer circumferential strain at the level of the papillary muscles; CSPMend—endocardium circumferential strain at the level of the papillary muscles; CSPMend-CSPMepi—the gradient between endocardium circumferential strain and epicardium circumferential strain at the level of the papillary muscles; CSAP—mid-layer circumferential strain at the apical level; CSAPend—endocardium circumferential strain at the apical level; CSAPend-CSAPepi—the gradient between endocardium circumferential strain and epicardium circumferential strain at the apical level.

Parameter	Coefficient	SE	OR	95% CI	AUC	*p*
GLS	0.32093	0.12197	1.3784	1.0853 to 1.7507	0.694	0.0085
GLSend	0.29082	0.10808	1.3375	1.0822 to 1.6531	0.700	0.0071
GLSend-GLSepi	0.55197	0.23152	1.73671	1.1032 to 2.7339	0.729	0.0067
CSMV	0.037002	0.089136	1.0377	0.8714 to 1.2358	0.526	0.6781
CSMVend	0.085917	0.072650	1.0897	0.9451 to 1.2565	0.590	0.2370
CSMVend-CSMVepi	0.183310	0.097038	1.2012	0.9931 to 1.4528	0.644	0.0589
CSPM	0.18874	0.10263	1.2077	0.9877 to 1.4768	0.650	0.0659
CSPMend	0.21235	0.079006	1.2366	1.0592 to 1.4437	0.705	0.0072
CSPMend-CSPMepi	0.22991	0.094868	1.2585	1.0449 to 1.5157	0.680	0.0154
CSAP	1.1634	0.078330	1.1634	0.9978 to 1.3564	0.629	0.0534
CSAPend	0.14511	0.062149	1.1562	1.0236 to 1.3059	0.659	0.0196
CSAPend-CSAPepi	0.18520	0.093819	1.2035	1.0013 to 1.4464	0.640	0.0484

**Table 8:** Stepwise multivariate regression analysis model in identifying STE parameters predictors of two years mortality in DCM patients. Data are presented as mean ± standard deviation. Abbreviations: DCM—dilated cardiomyopathy; SE—standard error; OR—odds ratio; CI—confidence interval; LAVi—left atrium volume indexed; CSPMend—endocardium circumferential strain at the level of the papillary muscles.

Parameter	Coefficient	SE	OR	95%CI	*p*
Hypertension = 0	1.61529	0.61661	5.0293	1.5019 to 16.8415	0.0088
LAVi	0.080420	0.025152	1.0837	1.0316 to 1.1385	0.0014
CSPMend	0.22247	0.089506	1.2492	1.0482 to 1.4887	0.0129

**Table 9:** AUC analysis of the stepwise multivariate regression model in identifying STE parameter predictors of two-year mortality in DCM patients.

Area Under the ROC Curve (AUC)	0.846
Standard Error	0.0456
95% Confidence interval	0.759 to 0.912

**Table 10:** **AUC analysis** to identify the sensitivity and specificity of the model, including CSPMend as an incremental predictor of two-year mortality in patients with DCM and HF.

Area Under the ROC Curve (AUC)	0.705
Standard Error	0.0618
95% Confidence interval	0.603 to 0.793
z statistic	3.308
Significance level *p* (Area = 0.5)	0.001
Associated criterion	**>−10.1**
Sensitivity	80.95
Specificity	60.53

**Table 12:** Intra-observer variability of multilayer strain. ICC—intra-class correlations; CI—confidence intervals; GLS—mid-layer longitudinal strain; GLSend-endocardium longitudinal strain; GLSend-GLSepi—the gradient between endocardium longitudinal strain and epicardium longitudinal strain; CSMV—mid-layer circumferential strain at the level of the mitral valve; CSMVend—endocardium circumferential strain at the level of the mitral valve; CSMVend-CSMVepi—the gradient between endocardium circumferential strain and epicardium circumferential strain at the level of the mitral valve; CSPM—mid-layer circumferential strain at the level of the papillary muscles; CSPMend—endocardium circumferential strain at the level of the papillary muscles; CSPMend-CSPMepi—the gradient between endocardium circumferential strain and epicardium circumferential strain at the level of the papillary muscles; CSAP—mid-layer circumferential strain at the apical level; CSAPend—endocardium circumferential strain at the apical level; CSAPend-CSAPepi—the gradient between endocardium circumferential strain and epicardium circumferential strain at the apical level.

Parameter	95% CI	ICC
GLS	0.821 to 0.988	0.952
GLSend	0.882 to 0.992	0.969
GLSend-GLSepi	0.794 to 0.986	0.945
CSMV	0.655 to 0.975	0.901
CSMVend	0.64 to 0.973	0.897
CSMVend-CSMVepi	0.566 to 0.966	0.871
CSPM	0.666 to 0.976	0.905
CSPMend	0.771 to 0.984	0.938
CSPMend-CSPMepi	0.636 to 0.973	0.895
CSAP	0.855 to 0.99	0.962
CSAPend	0.891 to 0.993	0.972
CDAPend-CSAPepi	0.858 to 0.991	0.963

## Data Availability

Data are available upon request in specific conditions.
